# Health data justice: building new norms for health data governance

**DOI:** 10.1038/s41746-023-00780-4

**Published:** 2023-02-28

**Authors:** James Shaw, Sharifah Sekalala

**Affiliations:** 1 Department of Physical Therapy, Temerty Faculty of Medicine, University of Toronto, 500 University Avenue, Toronto, ON M5G 1V7 Canada; 2 School of Law, University of Warwick, Coventry, CV4 7AL United Kingdom

**Keywords:** Medical ethics, Health policy

## Abstract

The retention and use of health-related data by government, corporate, and health professional actors risk exacerbating the harms of colonial systems of inequality in which health care and public health are situated, regardless of the intentions about how those data are used. In this context, a data justice perspective presents opportunities to develop new norms of health-related data governance that hold health justice as the primary objective. In this perspective, we define the concept of health data justice, outline urgent issues informed by this approach, and propose five calls to action from a health data justice perspective.

## Introduction

Advances in computational methods in the fields of Artificial Intelligence and Data Science have generated substantial attention to the ethical and social issues associated with health-related data. The value of these analytic methods is founded on persistent growth in the variety and volume of health-related data, driven by the growing global use of digital technologies to retrieve, record, and communicate health information^1,2^. Although accurate estimates of the global quantity of health-related data are virtually impossible to develop, larger volumes of data are generated each year, with annual global volumes likely to be in the trillions of bytes^1,3^. Data collected in contexts of public health and health care are those most obviously considered health-related data, but awareness is emerging about health-related uses of data collected outside of these contexts as well (e.g., through digital commerce or social media)^4^. The collection and use of these data present important social and ethical concerns. Alongside growing international recognition of the unwarranted influence of technology corporations in public health care systems^5,6^, and ongoing experiences of corporate and state colonialism in global health contexts^7,8^, these trends have raised the prominence of questions about the relationships between health equity, social justice, and digital data.

In this Perspective, we summarize the emerging literature on data justice in the context of health-related data and justify the importance of deeper attention to health data justice in particular. We present a definition of health data justice and outline a series of urgent issues for attention from a health data justice perspective. We conclude by presenting a series of new norms that need to be developed and present five calls to action for multiple stakeholder groups in health-related data science to support the implementation of a health data justice approach.

## What are health-related data?

The governance of health-related data and other personal data is evolving in important ways in jurisdictions around the world, closely connected to evolution in thinking about the definition and permissible uses of health data. In the United States of America and Canada, the category of health data continues to be defined as those data collected by certain actors specified in law who collect and use data in contexts directly linked to the delivery of health care and public health services^9^. However, these jurisdictions are contemplating a shift toward a more comprehensive definition of health data as expressed in the European General Data Protection Regulation (GDPR)^9^. For the GDPR, health-related data are referred to as “data concerning health” and are defined as “all data pertaining to the health status of a data subject which reveal information relating to the past, the current, or future physical or mental health status of the data subject”^10^. In our perspective, we are concerned with this broader category of “data concerning health”, which we refer to as “health-related data”, and maintain a special emphasis on data collected in contexts of health care and public health (the latter being referred to specifically as “health data” in contexts such as the United States of America).

The definition of health-related data provided by the GDPR represents an important departure from the source-specific definitions in other jurisdictions, because it includes data from any source and not only those collected for the express purpose of informing health care and public health services^11^. According to this definition, any data that can convey features of the health status of an individual can be considered health-related data. This broader definition of health-related data becomes complicated where existing governance mechanisms are unable to adequately account for analytic methods using diverse data sources to infer dimensions of health status^12^. One important example that is now well-established in academic literature is the use of social media data to infer insights about the mental health status of social media users^4^.

Some jurisdictions around the world are expanding their definitions similar to the GDPR. For example, an initiative to facilitate data sharing related to COVID-19 across nine African countries benchmarked their processes according to the standards set out in the GDPR^13^. Although not all jurisdictions are explicitly moving toward broader definitions of health-related data and stricter regulations on secondary uses, the shifting definition of health-related data and associated standards of regulation raises two important points that are noteworthy for the ensuing discussion. First, there is growing international recognition that broader definitions of what constitutes health-related data are necessary, given the growing capabilities of data analysts to establish health-related insights using a variety of data sources. Second, the stakeholders implicated in discussions of health-related data justice extend beyond healthcare providers, public health officials, and government actors to include commercial sector stakeholders such as social media and digital technology companies as well.

## What is health data justice?

Data justice refers to a group of frameworks informing the study and use of data in ways that prioritize the needs and experiences of structurally marginalized communities, and contribute to efforts to redress structural, institutional, and political injustices. Important contributions to the development of the data justice perspective have come from the fields of surveillance studies^14^, social justice^15^, global health^16^, Indigenous data sovereignty^17^, and black feminism^18,19^, and we refer readers to these foundational works for further detail on data justice more generally. Here we focus on the application of a data justice perspective to health-related data specifically.

Drawing on key contributors to social justice in the fields of philosophy and social theory^20–23^, and works on health justice^24,25^, we emphasize two related features of health data justice. First, we propose that health data justice situates equitable participation in health care and public health services as a fundamental organizing principle. Where groups are unable or unwilling to participate in health care and public health as a result of historical and ongoing oppressions, and are thereby excluded from the generation, collection, and use of data implied by that participation, injustices are conferred upon them. Conversely, where participation in systems of health care and public health and the data collection and uses that accompany that participation causes harm to groups, the same conclusion can be drawn. These realities represent the close links between participation in health care and public health on the one hand, and the generation and use of health-related data on the other.

Second, and related, health data justice emphasizes efforts to dismantle institutional obstacles that interfere with pursuing social justice in health care and public health. Building on the data justice literature referred to earlier, this approach brings attention away from specific techniques of data science or machine learning and the technical definitions of bias and fairness that accompany them, and toward the institutional aims and practices that provide a home for such techniques in the first place. The institutional focus raises the level of analysis to that of the social realities that frame the governance, goals, and deployment of health-related data science.

Health data justice, then, is an orientation to the study and use of health-related data in ways that aim to redress the exclusions of structurally marginalized communities from systems of health care and public health, the oppressions faced by communities when participating in such systems, and the institutions responsible for governing participation. Mapping on to these aims, a health data justice approach draws attention to a series of issues that demand attention and new norms for addressing them, which we turn to next. Importantly, when structures and strategies are implemented that achieve these aims, all members of a population benefit. Such approaches not only offer protections against a variety of potential harms across population groups, but also offer the potential of a more culturally safe, inclusive, trustworthy experience of health care and public health for all.

## Urgent issues for health data justice

The description of health data justice outlined here points to several issues that demand urgent attention if the governance of health-related data is to advance the aims of data justice. The list of issues presented in Table 1 involves practices related to the actors using health-related data and the communities affected by their use. Issues are not only specific to data collected in the context of health care and public health, but also in the use of non-health data to generate health-related insights. Some of these issues are local and others are international, illustrating the demand for coordinated governance approaches across political jurisdictions. Ultimately, the issues demonstrate the lack of attention in research, policy, and governance given to practices related to injustices of health data and strategies to actively promote health data justice.

**Table 1:** Urgent issues for health data justice.

Health Data Issue	Description
Health data colonialism	Actors leveraging power and resources to introduce digital technologies in relatively lower resource settings in order acquire data.
Commercial uses of health data	Commercial actors using health data to generate technologies used to produce commercial profits.
Health-related uses of non-health data	Actors generating health-related insights from data generated outside of health care or public health contexts.
Misplaced good intent	Actors engaging in uses of health data that cause unintended harm despite intentions to be helpful.
Justified mistrust	Community members mistrusting institutions to collect and use health data as a result of past and ongoing oppressions.
Group harms	The accrual of harms to particular communities as a result of uses of health data.
Data provenance and representation	The nature and quality of health data used to generate insights, including biases embedded in data and the exclusions of groups from datasets.
Exclusionary design	The design of data-intensive technologies based on datasets or design processes that exclude particular communities.
Systematic exclusion from datasets	The exclusion of particular communities or populations from datasets that are used to inform important health-related decisions.
Evolving harms of data uses	The observation that the harms of health data uses evolve over time, both in immediate consequences for affected communities and the erosion of agency to shape data uses in the longer term.
Techno-solutionism	The belief and set of practices in which technology can and should solve all problems, neglecting the social and institutional challenges at the root of many health-related issues.

Advancing a health data justice agenda requires both efforts to halt practices that perpetuate structural inequities and to promote practices that employ health data in service of enhancing the power, agency, and participation of structurally marginalized communities. In so doing, these practices serve to enhance equity in participation in health care and public health while building health systems that work better for entire populations. To inform these practices, health-related data science requires a data justice agenda when using health-related data.

## New norms and calls to action for health data justice

Historically, law, policy, and practice associated with health-related data have been based on a set of norms arising from conventional views of data as individually derived, owned by the collector, and subject to fragmented domestic policy restrictions. The GDPR has promoted changes to these norms by explicitly acknowledging the realities of contemporary data practices and prioritizing the rights of data subjects^26–28^. Advancing health data justice will require a further evolution of norms regarding health-related data and we propose that these new norms for health data justice constitute essential sites of collective scholarship and action for the future.

Table 2 presents an overview of five domains in which we propose new norms must develop to advance a health data justice agenda. These norms relate to data ownership (both individual and collective), institutional control, international cooperation, and public-private partnerships. The domains in which we propose to generate new norms are longstanding and deeply institutionalized in international policy environments and facilitating change on such a scale is an immense task. To accelerate engagement with the challenge of advancing these new norms, we propose five calls to action for researchers, clinicians, innovators, corporations, and data governance bodies representing practical steps toward health data justice.

1. Take historical marginalization seriously. Institutions of health care delivery, research, and innovation have harmed communities in important ways that generate mistrust over generations, and these histories must be understood to meaningfully work toward health data justice.
2. Build diverse knowledge and experience in health data governance. Commit to networking and collaborating with people who have different perspectives and life experiences than your own and engaging with disciplines (such as the social sciences) that can present different scholarly perspectives on data-intensive health innovation.
3. Build coalitions of action in partnership with community groups. Building trustworthy partnerships with community members who are affected by health-related data science requires an investment of time and energy over the longer term. Acknowledge the time necessary and build these investments into present and future planning. Where barriers exist to advancing projects based on a health data justice perspective, identify collaborators who can support the advancement of health data justice elsewhere.
4. Promote transnational regulatory cooperation for digital health governance. Invest in collaboration with stakeholders in other national jurisdictions to explore the implications of health data justice approaches to governance at the transnational level.
5. Invest in a health data justice approach to commercial partnerships. Commercial actors are essential stakeholders in health-related data science and encouraging deeper reflection among all team members on the implications of a health data justice perspective is necessary to advance this approach to governance in meaningful ways.

**Table 2:** New norms for health data justice.

Norms of individual ownership: Norms need to evolve to explicitly acknowledge and respect the individual as the ultimate owner of health-related data regardless of the context in which they were generated or collected, and regardless of the actor or entity currently in possession of the data.
Norms of collective ownership: Where a community is deemed an alternative owner of health-related data, further principles of community ownership need to be developed and recognized at an international level and by multiple actors in health data ecosystems. These should build on important successes in collective data ownership and governance, such as those generated by advocates of Indigenous data sovereignty.
Norms of institutional control: Where institutions such as healthcare organizations possess and control health-related data, the norms by which uses of those data are decided must shift toward greater community leadership.
Norms of international cooperation: Acknowledging that the collection and use of health-related data are international practices, norms of health data policy will need to align internationally to allow for greater protections for health-related data, especially for the most marginalized.
Norms of public-private partnership: Where private interests such as corporations are using health-related data to build products that will ultimately generate profits, business norms must evolve to seek out clearer permissions for uses of health data and ensure benefits of data uses to return to benefit structurally marginalized communities who generated the data in the first place.

## Conclusions

The growing capabilities of data science to harness insights for the improvement of health care and public health should be celebrated, but not at the expense of communities that have been marginalized by historical and contemporary practices of injustice. Given the collection of urgent issues identified by a health data justice perspective, we propose that deep change is necessary for a justice-oriented approach to the governance and use of health-related data. Adopting the calls to action outlined here and advancing new norms for health data justice will build a foundation for health data justice on a global scale, presenting a path for a socially just relationship between data science, public health, and health care.

## Data Availability

There are no data associated with this article.
